# Anti-Inflammatory Effects of Fatty Acid Amide Hydrolase Inhibition in Monocytes/Macrophages from Alzheimer’s Disease Patients

**DOI:** 10.3390/biom11040502

**Published:** 2021-03-26

**Authors:** Valerio Chiurchiù, Lucia Scipioni, Beatrice Arosio, Daniela Mari, Sergio Oddi, Mauro Maccarrone

**Affiliations:** 1 Institute of Translational Pharmacology, National Research Council (CNR), 00133 Rome, Italy; 2 Laboratory of Resolution of Neuroinflammation, European Center for Brain Research (CERC)/IRCCS Santa Lucia Foundation, 00143 Rome, Italy; 3 Laboratory of Neurochemistry of Lipids, European Center for Brain Research (CERC)/IRCCS Santa Lucia Foundation, 00143 Rome, Italy; l.scipioni@hsantalucia.it; 4 Geriatric Unit, Fondazione Ca’ Granda, IRCCS Ospedale Maggiore Policlinico, 20122 Milan, Italy; beatrice.arosio@unimi.it (B.A.); daniela.mari@policlinico.mi.it (D.M.); 5 Department of Clinical Sciences and Community Health, University of Milan, 20122 Milan, Italy; 6 Faculty of Veterinary Medicine, University of Teramo, 64100 Teramo, Italy; 7 Department of Biotechnological and Applied Clinical Sciences, University of L’Aquila, 67100 L’Aquila, Italy

**Keywords:** Alzheimer’s disease, cytokines, fatty acid amide hydrolase, immunomodulation, monocytes/macrophages

## Abstract

Growing evidence shows that the immune system is critically involved in Alzheimer’s disease (AD) pathogenesis and progression. The modulation and targeting of peripheral immune mechanisms are thus promising therapeutic or preventive strategies for AD. Given the critical involvement of the endocannabinoid (eCB) system in modulating immune functions, we investigated the potential role of the main elements of such a system, namely type-1 and type-2 cannabinoid receptors (CB_1_ and CB_2_), and fatty acid amide hydrolase (FAAH), in distinct immune cell populations of the peripheral blood of AD patients. We found that, compared to healthy controls, CB_1_ and CB_2_ expression was significantly lower in the B-lymphocytes of AD patients. Moreover, we found that CB_2_ was significantly lower and FAAH was significantly higher in monocytes of the same subjects. In contrast, T-lymphocytes and NK cells did not show any variation in any of these proteins. Of note, monocytic CB_2_ and FAAH levels significantly correlated with clinical scores. Furthermore, the pharmacological inactivation of FAAH in monocytes and monocyte-derived macrophages obtained from AD patients was able to modulate their immune responses, by reducing production of pro-inflammatory cytokines such as TNF-α, IL-6 and IL-12, and enhancing that of the anti-inflammatory cytokine IL-10. Furthermore, FAAH blockade skewed AD monocyte-derived macrophages towards a more anti-inflammatory and pro-resolving phenotype. Collectively, our findings highlight a central role of FAAH in regulating AD monocytes/macrophages that could be of value in developing novel monocyte-centered therapeutic approaches aimed at promoting a neuroprotective environment.

## 1. Introduction

Alzheimer’s disease (AD) is the most common form of dementia among people over the age of 65, accounting for 50–60% of all cases. Several theories have been proposed to explain AD etiology, and the amyloid-β peptide (Aβ) accumulation hypothesis has dominated the field for many years [1]. Yet, accumulated evidence suggests that AD pathogenesis and progression are not only restricted to the neuronal compartment, but they involve strong interactions with immunological mechanisms occurring both within the brain and at the periphery. For instance, misfolded Aβ42 and other aggregated proteins bind to pattern recognition receptors on the resident immune cells of the brain (i.e., microglia), and trigger an immune response characterized by the release of a plethora of inflammatory mediators that contribute to disease progression and severity. Furthermore, in the sporadic form of AD, the accumulation of Aβ42 is likely due to its defective clearance by microglial cells [2,3]. Recent research has shown that systemic immune dysfunctions are also critically associated with AD pathology. The latter includes changes in the distribution and activation of specific innate and adaptive immune cell populations, or their infiltration in the brain, thus further sustaining and exacerbating neuroinflammation (3). Modulation and targeting of immune mechanisms are now considered promising therapeutic/preventive strategies for AD [4].

In this context, several in vitro and in vivo studies have suggested that the endocannabinoid (eCB) system plays a critical pathophysiological role in several neuroinflammatory and neurodegenerative diseases, including AD [5,6]. In particular, perturbations in type-1 and type-2 cannabinoid receptors (CB_1_ and CB_2_) as well as in the main enzyme that terminates eCB signaling (i.e., the fatty acid amide hydrolase (FAAH); EC 3.5.1.99) have been reported in most brain regions that are involved in AD, as well as in the peripheral blood of AD patients [5,6,7]. This evidence led to a plethora of preclinical studies in various experimental models of AD, aimed at investigating the effects of specific cannabis extracts, selective CB_1_/CB_2_ agonists, or specific inhibitors of eCB metabolic enzymes (as extensively reviewed in references [5,6,7,8]. For instance, robust evidence reported that the pharmacological and genetic inactivation of FAAH not only restored the levels of its endogenous substrate anandamide (*N*-arachidonoylethanolamine), but it also dramatically ameliorated AD-like symptoms in terms of Aβ accumulation, neuroinflammation and cognitive decline [9,10]. Remarkably, the phenotypical characterization of AD-related changes in human patients revealed that FAAH is specifically upregulated in peripheral blood mononuclear cells [7] and in the hippocampus and entorhinal cortex [11], suggesting a prominent role of this enzyme in human AD. Accordingly, it has been found that the FAAH substrate anandamide is reduced in the mid-frontal and temporal cortex of AD patients, where it is inversely correlated with Aβ42 content [12]. Hence, given the critical involvement of the eCB system in AD and in modulating the responses of the majority of immune cells [13,14], here we sought to investigate whether a possible correlation could exist between the molecular changes of the eCB system in peripheral blood immune cells and AD, and whether targeting altered eCB system elements could exert anti-inflammatory actions. In particular, the present study was established as a follow up of our previous evidence on increased FAAH expression in the peripheral blood mononuclear cells (PBMCs) of AD patients [7], in order to unravel which immune cell population is responsible for such change. Here we showed that FAAH expression is significantly higher in the monocytes of AD patients, and that its pharmacological inhibition in monocytes and monocyte-derived macrophages modulates the reduction in pro-inflammatory cytokines and subverts the immunophenotype of M1-like macrophages towards a more pro-resolving one.

## 2. Materials and Methods

### 2.1. Study Participants and Sample Collection

Peripheral blood from age and sex-matched control subjects (CT, *n* = 15) (9 females and 6 males, mean age = 61.3 ± 5.2) and patients with Alzheimer’s disease (AD, *n* = 15) (10 females and 5 males, mean age = 65.7 ± 4.8) were recruited and collected at the Geriatric Unit of the Policlinico Hospital in Milan, Italy. Data about their medical history, physical and neurological examination, neurocognitive evaluation (mini-mental state examination), computed tomography or MRI scan, and screening laboratory tests consisting of the assessment of tau, phospho-tau (p-tau), and Aβ42 levels by ELISA (Innogenetics) were collected. AD patients fulfilled the Dubois et al. criteria [15]. Control subjects were examined likewise to exclude the presence of neurological and cognitive disorders.

### 2.2. Cell Preparation

PBMCs were isolated after venous puncture from CT and AD patients and were separated by density gradient over Ficoll-Hypaque (Pharmacia, Uppsala, Sweden) according to standard procedures [16]. In particular, 10 mL of peripheral blood was diluted 1:1 with sterile PBS and gently layered on the top of 15 mL of Ficoll and centrifuged for 30 min at 100× *g* at 4 °C in a swing-out bucket with no brake. The buffy coat containing the PBMCs formed in the interphase between the Ficoll and plasma was gently collected with a sterile Pasteur pipette and washed twice in sterile PBS for 10 min at 4 °C, and resuspended in RPMI 1640 complete medium supplemented with 10% (*v*/*v*) heat-inactivated human serum before further processing. For flow cytometry evaluation of CB_1_, CB_2_ and FAAH expression, 2–3 × 10^6^ cells were counted and stained as described in Section 2.4.

For functional studies on the role of the FAAH inhibitor, 5–6 × 10^6^ PBMCs were used to isolate CD14+ monocytes in order to differentiate them into macrophages, as described in Section 2.3.

### 2.3. Monocyte Activation and Macrophage Polarization

CD14+ monocytes were purified by means of cell sorting and were left untreated or were pretreated with URB597 for 30 min. To allow cytokine synthesis, the monocytes were then stimulated with 100 ng/mL of LPS (Sigma-Aldrich, St. Louis, MO, USA) for 5 h in the presence of 10 μg/mL of brefeldin A (Life Technologies, Carlsbad, CA, USA) and cytokine production was evaluated by flow cytometry, as reported [17]. For macrophage polarization, the CD14+ monocytes were left in adhesion for 2 h in complete RPMI 1640 medium in order for them to adhere. After two hours, non-adherent cells were removed and adhering monocytes were gently rinsed with PBS and cultured in fresh complete medium supplemented with 50 ng/mL of M-CSF (Miltenyi Biotec, Bergisch Gladbach, Germany) for M0 (homeostatic macrophages) polarization for 6 days. At day 2 and 4, cells were provided with new supplemented medium, as reported [18]. At day 6, cells were rinsed with PBS and polarized into M1 (pro-inflammatory macrophages) in the presence of 100 ng/mL of LPS and 10 ng/mL of mouse IFN-γ (Miltenyi Biotec, Bergisch Gladbach, Germany). Adherent M1 macrophages were collected following the addition of trypsin-EDTA solution, stained and analyzed for immunophenotyping by flow cytometry, as detailed in Section 2.4.

### 2.4. Flow Cytometry

Freshly isolated PBMCs (2 × 10^6^ cells) were washed twice with PBS and stained with previously validated [17] rabbit anti-CB_1_ and anti-CB_2_ (Cayman Chemicals, Ann Arbor, MI, USA) antibodies, or with goat anti-FAAH (Santa Cruz, Santa Cruz, CA, USA) antibodies in stain buffer (PBS-supplemented with 0.5% FCS and 0.02% NaN_3_) for 30 min at 4 °C. Cells were then washed with PBS (1X) and stained with anti-rabbit or anti-goat Alexa-633 secondary antibody (1:100) and a combination of fluorescence-conjugated antibodies against markers of specific immune cell populations for 15 min in the staining buffer at 4 °C. Cells were also stained with respective isotypes and the Alexa-633 secondary antibody alone in order to assess background staining and specificity. For the evaluation of intracellular cytokine production in monocytes, at the end of the incubation, cells were fixed with 4% paraformaldehyde for 10 min, and then stained intracellularly with PE-conjugated anti-TNF-α, FITC-conjugated anti-IL6, APC-conjugated anti-IL12p40 and v450-conjugated anti-IL10 antibodies in 0.5% saponin at room temperature for 30 min. For the evaluation of monocytes or macrophage activation markers, cells were stained at cell surface with Cy-Chrome-conjugated anti-CD80 or FITC-conjugated anti-CD69, APC-conjugated anti-CD86, and PE-conjugated anti-CD206 antibodies. The details of all the antibodies used for surface and intracellular staining, including their dilutions and manufacturers, are reported in Table 1.

**Figure 1:**
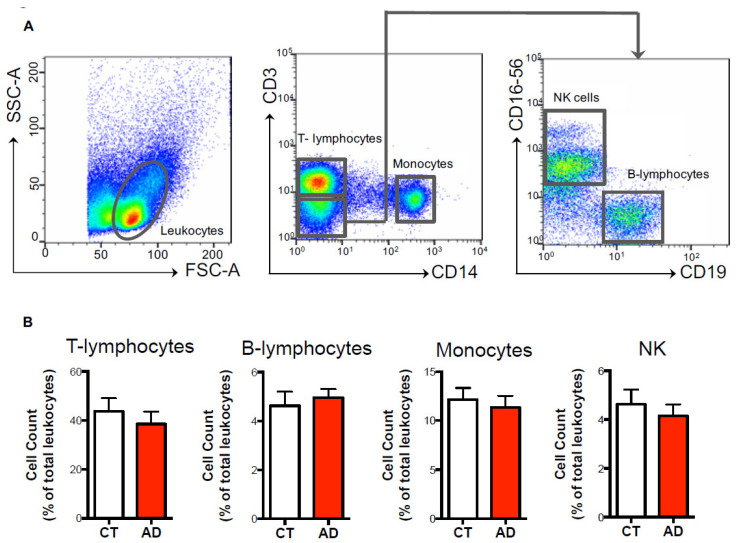
Immunophenotyping of immune cell populations. PMBCs (2–3 × 10^6^) were stained at the cell surface with specific markers for the main innate and adaptive immune cell populations, and were analyzed through polychromatic flow cytometry, as detailed in the materials and methods. (**A**) According to physical parameters, total leukocytes were identified and further gated as CD14+ (monocytes) or CD3+ (T-lymphocytes) upon exclusion of CD3- cells. CD3- cells were subsequently gated against CD19+ (B-lymphocytes) and CD16+CD56+ (NK cells). (**B**) Histograms of the cell counts (expressed as percentage ± s.e.m. of total leukocytes) of T-lymphocytes, B-lymphocytes, monocytes and NK cells in both healthy controls (CT) and Alzheimer’s disease (AD) subjects.

For the evaluation of the expression of CB_1_, CB_2_ and FAAH in the different immune cell subsets, a total of 2–3 × 10^6^ cells was acquired on the 3-laser and 9-fluorescences flow cytometer FACS Cyan (Beckman Coulter, Chaska, MN, USA). The total cells were plotted against physical parameters of side scatter (SSC-A) and forward scatter (FSC-A), and a first gate was drawn on total leukocytes, excluding debris on the left angle of the plot. Subsequently, we plotted CD14+ cells against CD3+ and the following 3 different populations were observed: CD14+ cells (monocytes), CD3+ cells (T-lymphocytes) and CD3- cells (remaining leukocytes). We further gated on the CD3- cells and these were plotted against the CD19+ cells (B-lymphocytes) and CD56+CD16+ (NK cells) (Figure 1A).

**Figure 2:**
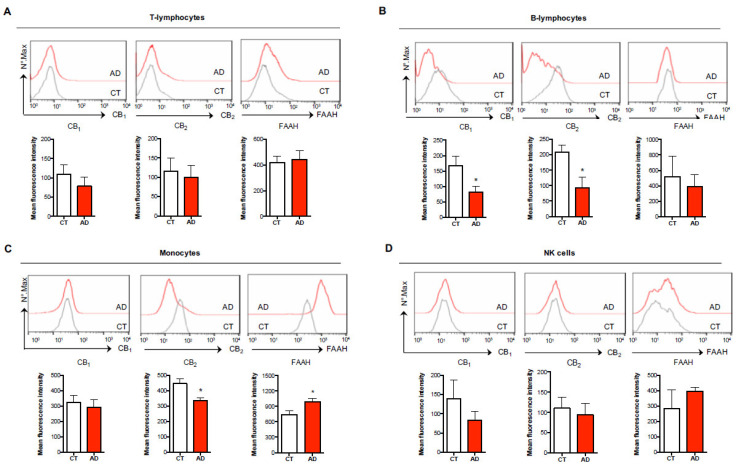
Characterization of endocannabinoid (eCB) system elements in different immune cell subsets of healthy controls and AD subjects. Surface expression of type-1 and type-2 cannabinoid receptors (CB_1_, CB_2_) and intracellular expression of fatty acid amide hydrolase (FAAH) in human T-lymphocytes (CD3+) (**A**), B-lymphocytes (CD19+) (**B**), monocytes (CD14+) (**C**), and NK cells (CD56+) (**D**) of healthy controls (CT) and AD subjects was assessed through polychromatic flow cytometry as detailed in the materials and methods. The histograms represent the mean intensity fluorescence (MIF) ± s.e.m. of 7–9 independent experiments. * *p* < 0.05 vs. CT.

**Figure 3:**
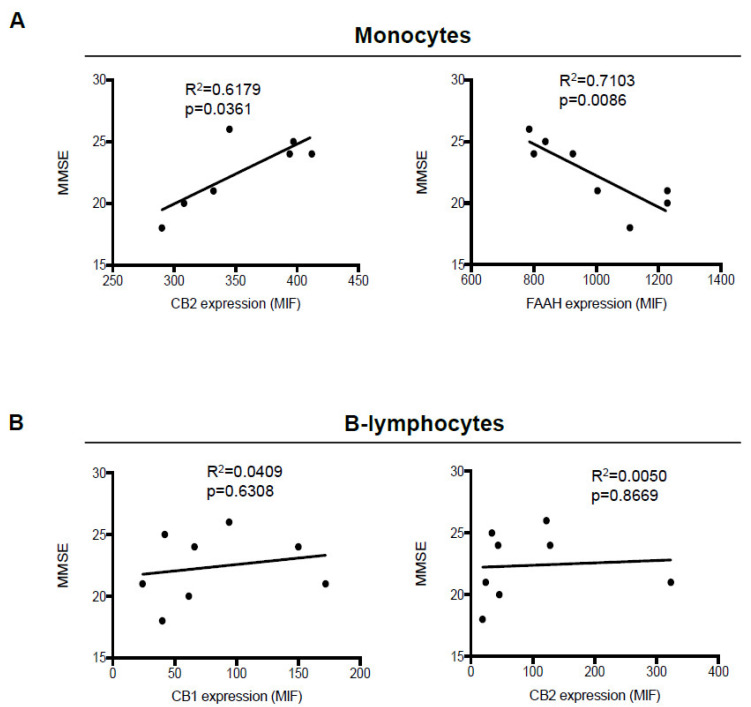
Correlation between mini-mental state examination (MMSE) scores and expression of eCB system members in monocytes and B-lymphocytes. Correlation plots between MMSE scores and expression (mean intensity fluorescence (MIF) values) of FAAH and CB_2_ receptors in monocytes, (**A**) and of CB_1_ and CB_2_ receptors in B-lymphocytes (**B**) from AD patients. Data were compared by linear regression analysis (*p* < 0.05).

This approach allowed us to simultaneously discriminate these 4 immune cell populations in a single sample and to both calculate their percentage among the total leukocytes (Figure 1B) and to analyze the expression of each eCB element (CB_1_, CB_2_ and FAAH) in each cell subset (Figure 2 and Figure 3).

The expression of CB_1_, CB_2_ and FAAH was shown by graphing the mean fluorescence intensity in each cell subset according to the standard flow cytometry procedure.

**Figure 4:**
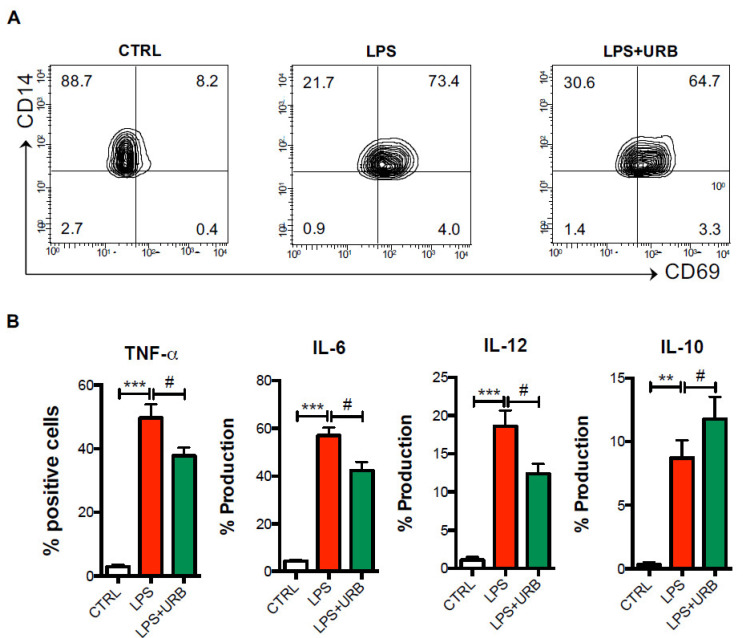
Pharmacological FAAH inhibition reduces monocyte inflammatory responses in AD patients. Cells from AD patients were cultured in the absence or presence of the FAAH inhibitor URB597 (URB), and then were challenged with LPS for 6 h in the presence of the exocytosis inhibitor brefeldin A as detailed in the materials and methods. By means of polychromatic flow cytometry, the surface expression of the activation marker CD69 (**A**) and intracellular production of TNF-α, IL-6, IL-12p40 and IL-10 (**B**) was measured. Data are shown as mean percentages of positive cells ± s.e.m. of 4–5 independent experiments. ** *p* < 0.01 and *** *p* < 0.005 vs. CTRL; # *p* < 0.05 vs. LPS.

**Figure 5:**
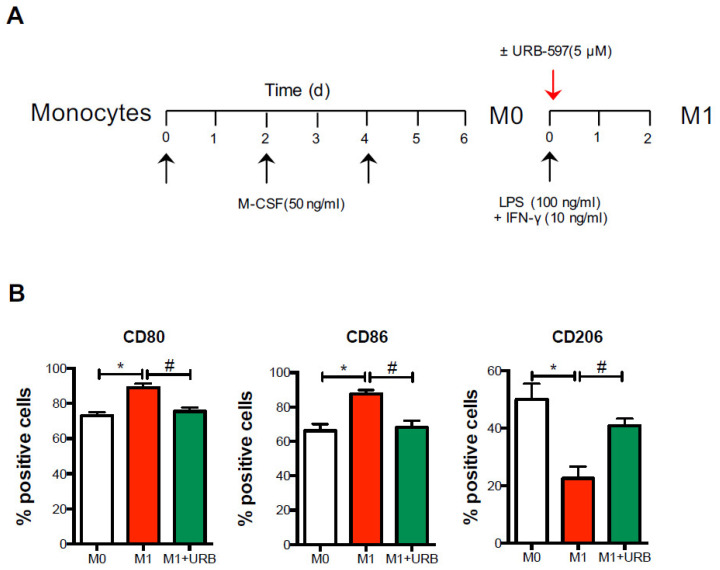
Pharmacological FAAH inhibition shifts M1 polarization. Cells from AD patients were differentiated into macrophages (M0) for 6 days in the presence of M-CSF, and subsequently polarized in M1 macrophages upon incubation with LPS and IFN-γ, in the absence or presence of the FAAH inhibitor URB-597 (URB) for 48 h. (**A**) By means of polychromatic flow cytometry, the surface expression of the polarization markers CD80, CD86 and CD206 was measured (**B**) as detailed in the materials and methods. Data are shown as mean ± s.e.m. of 4–5 independent experiments. * *p* < 0.05 vs. M0; # *p* < 0.05 vs. M1.

For the analysis of intracellular cytokines on purified CD14+ monocytes (Figure 4) and of activation markers on monocyte-derived macrophages (Figure 5), the no gating strategy was performed because in these experiments a homogeneous and pure population is present and directly stained with fluorochrome-conjugated antibodies against cytokines and activation markers.

For these experiments, 1–3 × 10^5^ cells were acquired on the same flow cytometer FACS Cyan, and the expressions of intracellular cytokines on monocytes and of activation markers on macrophages were shown by graphing the percentages of cells positive for each marker. Before analysis, each fluorescence was properly compensated and a compensation matrix was applied to all acquired samples to avoid any spill over.

### 2.5. Statistical Analysis

All data were expressed as means ± s.e.m. The differences between the two groups were compared using the non-parametrical Mann–Whitney U test, whereas differences between multiple groups were compared by one-way ANOVA followed by the Tukey test. All statistical analyses were performed with Prism 6.0 (GraphPad Software, San Diego, CA, USA) and *p*-values < 0.05 were considered statistically significant. Flow cytometry analysis was performed using the Flowjo analysis program (Treestar, Ashland, OR, USA).

## 3. Results

### 3.1. Differential Expression of Key Members of the eCB System in Peripheral Blood Cells of AD Patients

Initial studies were performed to assess whether AD patients showed any changes in the following main and most represented cell subsets of human peripheral blood: total CD3^+^ T-lymphocytes, CD14^+^ monocytes, CD19^+^ B-lymphocytes, and CD56^+^ NK cells. To this aim, polychromatic flow cytometry and several antibodies (Table 1) were used to correctly identify each cell population through the gating strategy shown in Figure 1A. Such immunophenotypic characterization revealed that the AD patients had similar percentages in all the innate and adaptive immune cell subsets compared to the healthy controls (CD3^+^ T-lymphocytes: 38.54 ± 5.08 % vs. 43.74 ± 5.35 %; CD14^+^ monocytes: 11.33 ± 1.19 % vs. 12.16 ± 1.69 %; CD19^+^ B-lymphocytes: 4.95 ± 0.35 % vs. 4.63 ± 0.57 %; CD56^+^ NK cells: 4.15 ± 0.47 % vs. 4.62 ± 0.59 %) (Figure 1B), indicating that during AD the distribution of the immune cell population remains unchanged.

Subsequently, we questioned whether each of these immune cell subsets differentially expressed the members of the eCB system that are particularly involved in the modulation of immune responses, namely CB_1_ and CB_2_ receptors and the main eCB-metabolizing enzyme FAAH. As reported in Figure 2, surface and/or intracellular staining showed that both T-lymphocytes and NK cells showed similar mean fluorescence intensity (MFI) levels in the expression of CB_1_, CB_2_ and FAAH between healthy and AD patients (Figure 2A,D), whereas B-lymphocytes showed no variation in FAAH expression but a significant reduction in both CB_1_ (MFI = 81.19 ± 19.02 vs. 167.30 ± 30.85) and CB_2_ (MFI = 92.50 ± 36.18 vs. 207.90 ± 23.14) receptors in AD patients (Figure 2B). Interestingly, when analyzing these eCB members in monocytes, the immunophenotyping not only revealed that CB_2_ expression was significantly reduced in AD patients compared to healthy controls (MFI = 334.40 ± 18.31 vs. 445.30 ± 30.38), with the levels of CB_1_ remaining unchanged (Figure 2C), but also that FAAH expression was significantly increased (MFI = 989.40 ± 64.53 vs. 742.30 ± 69.85). Of note, we observed that the expression levels of the different eCB members were much higher in monocytes compared to all the other immune cell populations, their levels being 2- to 3-fold higher than those found in T- and B-lymphocytes and in NK cells. These findings suggest that monocytes are by far the most responsive immune cells to endogenous eCBs in peripheral blood.

We next sought to determine whether the significant changes observed in CB_1_ and CB_2_ expression in B-lymphocytes, and of CB_2_ and FAAH in monocytes, correlated to the neurological and neurocognitive severity of AD patients, assessed through mini-mental state examination (MMSE) scores, where higher MMSE scores are associated with milder dementia. Although we did not observe any significant correlation of CB_1_ and CB_2_ receptors with MMSE scores in B-lymphocytes (Figure 3B), instead, in monocytes, the expression of CB_2_ showed a positive correlation (*p* = 0.0361), and higher levels were observed in the patients showing progressively higher scores. In contrast, FAAH expression showed a negative correlation (*p* = 0.0086), inasmuch as its levels progressively increased along with disease severity (Figure 3A).

### 3.2. Role of FAAH in Monocytes

Since the monocytes expressed remarkable levels of FAAH, which were even higher in AD patients and indicated their higher ability to metabolically inactivate endogenous eCBs, we next sought to investigate the possibility of pharmacologically inhibiting enzyme activity in the monocytes of AD patients with URB597, and to evaluate the inflammatory responses following LPS activation. As expected, compared to resting monocytes, LPS-activated monocytes showed significantly higher levels of the CD69 activation marker, and produced high levels of the pro-inflammatory cytokines TNF-α, IL-6 and IL-12, as well as of the anti-inflammatory cytokine IL-10. However, when incubated with URB597, both the expression of CD69 and the production of pro-inflammatory cytokines were significantly reduced (Figure 4A,B).

In particular, FAAH inactivation by URB597 induced a significant ~25% decrease in TNF-α and IL-6 production and a ~35% decrease in IL-12 (Figure 3B) compared to LPS-activated monocytes. Additionally, treatment with URB597 significantly increased IL-10 production by ~35% in LPS-activated monocytes (Figure 3B).

### 3.3. Role of FAAH in Macrophages

Since monocytes are only found in peripheral blood, and during inflammation they enter tissues and herein develop into macrophage subsets (which are represented by the classically activated M1 and alternatively activated or pro-resolving M2 macrophages), we next assessed whether FAAH inhibition was also able to impact macrophage polarization (Figure 5A).

As expected, and as already reported [18,19], M1 macrophages exhibited high levels of M1-like CD80 and CD86 markers and a low M2-like CD206 marker compared to the resting and non-polarized M0 macrophages. Interestingly, when URB597 was added during M1 polarization, the cells showed significantly lower levels of the M1-like markers CD80 and CD86 and higher levels of the signature M2-like marker CD206 (Figure 5B), suggesting that pharmacological inhibition of FAAH promoted an M1-to-M2 switch in AD.

## 4. Discussion

This is the first study reporting a detailed characterization of the expression of key members of the eCB system in distinct and specific cell populations of peripheral blood in AD patients compared to healthy donors. Such a study revealed a significant increase in FAAH only in the monocytes of AD patients and we uncovered its specific anti-inflammatory role in monocyte-derived macrophages.

Although the expression and function of CB_1_ and CB_2_ receptors, and of the eCB-metabolizing enzyme FAAH, has been widely studied in whole peripheral blood cells of several neurodegenerative diseases [5,6,20], as of yet no studies have addressed their expression within the different subsets of the immune system.

In the attempt to shed some light on the mononuclear cells of innate and adaptive immunity, which could express selected members of the eCB system, we performed polychromatic flow cytometry that allowed simultaneous interrogation of CB_1_, CB_2_ and FAAH expression in distinct cell populations. Such a multiparametric approach revealed that both CB_1_ and CB_2_ receptors and FAAH are far more abundant in innate (i.e., monocytes) rather than adaptive (i.e., lymphocytes) immune cells, suggesting that their function might be associated with the regulation of innate immune responses. In particular, our observed increase in FAAH in the monocytes of AD patients not only corroborates previous findings that reported an increase in this enzyme due to an altered epigenetic regulation of its gene promoter in total peripheral blood mononuclear cells of late-onset AD patients [7], but extends those findings and identifies monocytes as the immune cell population mostly responsible for reducing the eCB tone in AD. Consequently, adequate immune regulation by eCBs is prevented. This is particularly important since monocytes are by far the most represented immune cells infiltrating the brain of AD patients [21,22], and their macrophage progeny plays a critical role in orchestrating clearance and immune defense against misfolded and aggregated proteins [23]. Furthermore, the ability of a selective FAAH inhibitor to reduce the pro-inflammatory cytokine production from monocytes, and to partially subvert the immunophenotype of M1-like macrophages towards a more tolerogenic one, seems noteworthy, and might be promising in ultimately controlling the severity of neuroinflammatory processes. Although this requires validation in a larger cohort of AD patients and further experiments are needed to better understand the molecular mechanism of such an immunological switch, perhaps due to an increase in CB_2_ expression or activation of specific transcriptional pathways, our findings suggest that FAAH might also be an ideal target for other neuroinflammatory and neurodegenerative diseases where innate immune cells are strongly implicated in their immunopathogenesis, such as multiple sclerosis and Parkinson’s disease. Additionally, despite this study being the first to investigate the expression of several elements of the eCB system within the different immune cell populations, the expression of other related eCB receptors or enzymes such as GPR55 and GPR18 or MAGL and DAGL remains to be investigated. Lastly, our results suggest that it could be useful to explore the potential role of synthetic compounds in which FAAH inhibition is combined with a simultaneous activation of CB_2_, opening new insights for multitargeting therapeutic strategies.

## 5. Conclusions

Overall, our findings provide a better understanding of the role of monocytes/macrophages in AD immunopathogenesis, corroborating the hypothesis that peripheral immune cells play a crucial role in this disease. Although it seems necessary to further explore the role of FAAH in controlling monocyte/macrophage functions during AD, along with the underlying molecular mechanisms, this study unfolds new opportunities for a FAAH-oriented therapy targeting specific cells of the innate immune system.

## Figures and Tables

**Table 1:** Antibodies used for the immunophenotypic characterization of PBMCs and for the evaluation of cytokines and inflammatory markers in monocytes/macrophages.

Antibody	Manufacturer	Dilution
CD3 FITC	eBioscience	1:60
CD14 Brilliant Violet	Biolegend	1:100
CD19 APC	Miltenyi biotec	1:100
CD16 PE	eBioscience	1:100
CD56 PE	Beckman Coulter	1:100
CD69 FITC	Biolegend	1:80
CD80 Cy-Chrome	Biolegend	1:800
CD86 APC	Biolegend	1:100
CD206 PE	Beckman Coulter	1:80
TNF-a PE	Biolegend	1:100
IL-6 FITC	Biolegend	1:100
IL-12 p40 APC	Miltenyi biotec	1:60
IL-10 v450	BD Pharmingen	1:50

## Abbreviations

AD	Alzheimer’s disease
CB1	Cannabinoid receptor type-1
CB2	Cannabinoid receptor type-2
eCB	Endocannabinoid system
FAAH	Fatty acid amide hydrolase
IL-6	Interleukin-6
IL-12	Interleukin-12
IL-10	Interleukin-10
INF-g	Interferon-g
LPS	Lipopolysaccharide
MMSE	Mini-mental state examination
MRI	Magnetic resonance imaging
M-CSF	Macrophage colony stimulating factor
MFI	Mean fluorescence intensity
NK	Natural killer cells
PBMC	Peripheral blood mononuclear cell
TNF-a	Tumor necrosis factor-a
